# Estrogen Receptors in Colorectal Cancer: Facts, Novelties and Perspectives

**DOI:** 10.3390/curroncol28060361

**Published:** 2021-10-20

**Authors:** Ilaria Ditonno, Giuseppe Losurdo, Maria Rendina, Maria Pricci, Bruna Girardi, Enzo Ierardi, Alfredo Di Leo

**Affiliations:** 1 Section of Gastroenterology, Department of Emergency and Organ Transplantation, University “Aldo Moro” of Bari, 70124 Bari, Italy; ilaria.ditonno@gmail.com (I.D.); giuseppelos@alice.it (G.L.); mariarendina@virgilio.it (M.R.); ierardi.enzo@gmail.com (E.I.); 2 Ph.D. Course in Organs and Tissues Transplantation and Cellular Therapies, Department of Emergency and Organ Transplantation, University “Aldo Moro” of Bari, 70124 Bari, Italy; 3 THD SpA, 42015 Correggio, Italy; mirellapricci@libero.it (M.P.); brunagirardi@virgilio.it (B.G.)

**Keywords:** colorectal carcinoma, adenoma, polyps, chemoprevention, estrogens, estrogen receptors

## Abstract

Colorectal cancer (CRC) is the second cause of cancer-related death in both sexes worldwide. As pre-menopausal women are less likely to develop CRC compared to age-matched men, a protective role for estrogens has been hypothesized. Indeed, two isoforms of nuclear estrogen receptors (ER) have been described: ERα and ERβ. While the binding of 17beta-estradiol to ERα activates anti-apoptotic pathways, the interaction with ERβ activates caspase-3, inducing apoptosis. In this regard, several pieces of evidence show that ERβ tends to be under-regulated in advanced adenomas and CRC, with an opposite trend for ERα. Furthermore, ERβ stimulation slows adenomatous polyp growth and modulates relevant CRC pathways. Based on such considerations, dietary modulation of ER is promising, particularly in subjects with genetic predisposition for CRC. Nevertheless, the main limitation is the lack of clinical trials on a large population scale.

## 1. Introduction: The Burden of Colorectal Cancer (CRC) and Epidemiology in Women

Despite recent advances in screening and treatment, colorectal cancer (CRC) remains the second most common cause of cancer-related death in both sexes worldwide. CRC may grow as a vegetating mass, protruding in the lumen, or deeply in the intestinal wall, thus causing stenosis of the lumen. Adenocarcinoma is the most common histotype, and it is usually the result of adenomatous polyp degeneration. The staging of CRC is commonly performed using the American Joint Committee on Cancer (AJCC) or the Union for International Cancer Control (UICC) [1]. It was estimated that in 2020 approximately 147,950 individuals were diagnosed with CRC in United States and 53,200 did not survive the disease [2]. Moreover, a yearly 2% increase in CRC incidence has been observed in individuals younger than 50 years [3]. Potentially preventable risk factors, such as the highly processed Western diet, cigarette smoking and obesity, still play a relevant role in cancer development [4]. As for the non-modifiable risk factors, male gender confers an higher risk for both colorectal polyps and tumors, compared to the females, with odds ratios (OR) of 1.52 and 1.43, respectively [5]. Exposure to sex hormones, especially estrogen, has been proposed as a possible explanation for this gender-based difference, especially in relation to the different CRC risk seen in pre-menopausal versus post-menopausal women. According to the Women’s Health Initiative, the first subgroup is 40% less likely to develop CRC compared to age-matched men [6]. Conversely, the fall of estrogen levels seen in post-menopausal women may apparently explain the worse overall survival seen in this cohort compared to age-matched men after the diagnosis of metastatic CRC [7]. Similar findings were revealed following inflammatory bowel disease patients for 10 years: men had a 60% higher risk of developing CRC compared to women [8], suggesting a possible role of estrogen as a modulator of inflammation-related carcinogenesis. On the other hand, hormone replacement therapy may reduce the risk of CRC, as evidenced recently in a Swedish study, with a reduction of about 25% for current estradiol and estriol users [9].

## 2. Estrogen and Estrogen Receptor in CRC

Estrogen and estrogenic compounds, beyond their renowned role in the female reproductive system function and maturation, are involved in the physiological and pathophysiological mechanisms of other tissues, including the gastrointestinal system [10]. Two nuclear estrogen receptors (ER), ERα and ERβ, have been reported. ERs mediate genomic effects of estrogen after dimerization and translocation to the nucleus upon ligand binding, thus regulating the transcription of target genes [11]. Estrogen’s role in cell cycle regulation has been investigated by Acconcia et al. [12]; the binding of 17beta-estradiol (E2) to ERα activates anti-apoptotic pathways through ERK/MAPK and PI3K/AKT signaling. Contrarily, E2-ERβ interaction activates caspase-3, cleaving PARP and inducing apoptosis. Palmitoylation is suggested to be the molecular mechanism that allows ERα localization in plasmatic membrane and its activation of rapid non-genomic cell cycle proliferation signals [13]. ERβ, additionally, undergoes palmitoylation, inducing opposite effects and counteracting E2-ERα interaction with a pro-apoptotic effect.

**Figure 1:**
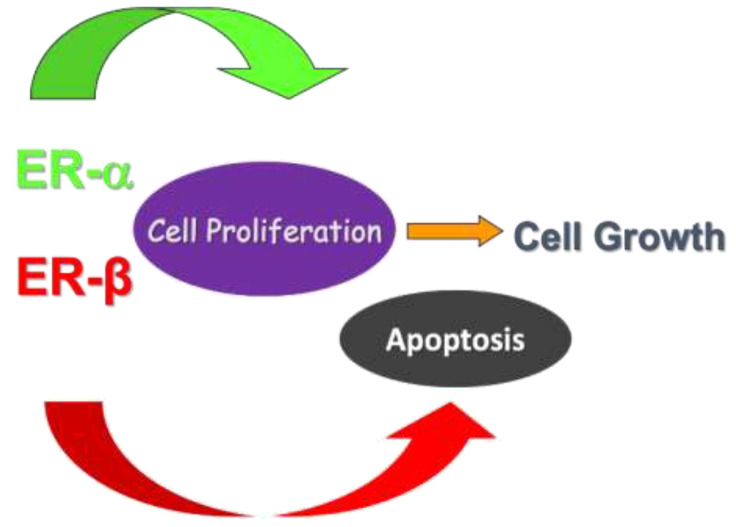
Evidence from the literature shows that the stimulation of ERα leads to activation of pathways involved in cell proliferation. On the other hand, ERβ promotes cell apoptosis in colon, thus inhibiting cell replication and tumoral growth.

ERα and ERβ are both expressed in the normal colorectal tissue, with a predominance of ERβ; however, upon the development of both adenomas and CRC, a shift in the ratio between the two has been registered, i.e., downregulation of ERβ and increase of ERα. ER expression has been studied in adenomatous tissue and compared to the normal colonic mucosa in patients with colonic polyps versus controls: a sharp reduction in ERβ with a parallel increase in cellular proliferative activity was demonstrated in colorectal adenomas compared to normal colonic tissue [14], thus highlighting a potential role of estrogen in the early phase of carcinogenesis (Figure 1). In sporadic polyps, a progressive decrease in the expression of ERβ isoforms ERβ1 and ERβ5 was observed in another study [15], showing also that the loss of ERβ expression was an independent predictor of cancer recurrence, while ERβ1 and ERβ5 were linked to improved survival. Additionally, the loss of expression of ERβ1 was correlated with mucinous adenocarcinoma [16], while a retained expression of ERβ was associated with a 50% reduction of overall mortality and a 76% decrease in cancer recurrence [17]. The evidence for ERα supporting carcinogenesis is less strong than for ERβ. Xie et al. [18] found that the expression rate of ERα mRNA in CRC tissue and corresponding normal colon tissue was 25% and 16.6%, respectively. Similarly, in another paper, ERα immunoreactivity was demonstrated only in 7% of samples [19]. However, there is some evidence that ERα promotes cancer cell migration [20], thus supporting metastatic spread, and that CRC overexpressing ERα has worse prognosis [21].

The evaluation of ER expression in tissue samples of six patients with familial adenomatous polyposis (FAP) after colectomy showed that ERβ was significantly reduced in both dysplastic adenomatous and carcinomatous tissues compared to normal colonic mucosa [22]. Interestingly, the ERβ expression in polyps arising in FAP patients is lower than in sporadic polyps [23]. Even in duodenal polyps of FAP patients, the same trend was observed. The progression of dysplasia paralleled an increase in ERα and Ki67 proliferation index as well a decrease in ERβ and apoptotic activity [24].

The chronic inflammatory environment seen in inflammatory bowel disease predisposes to CRC development; the entity of inflammation bears a higher risk of colonic malignant transformation, as stated by current guidelines, which recommend endoscopic surveillance for CRC screening in IBD [25]. It has been discovered that in this setting of CRC, ER may play a role. Indeed, E2 reduced inflammation in wild type mice treated with a pro-inflammatory agent, while mice knockout for ERβ showed increased proliferation of colonic cells [26]. ERβ stimulation even improved colitis as demonstrated in a murine model of dextran sulfate sodium (DSS)-induced colitis, where ERB041 (an ERβ-specific agonist) led to suppression of CD4+CD25− and CD8+ T lymphocytes and restoration of Tregs balance [27]. Colorectal tissue samples from long-standing ulcerative colitis (UC) patients have been retrospectively examined, stratified according to the level of tissue dysplasia, from non-dysplastic UC to colitis-associated carcinoma, and a progressive reduction of ERβ expression was registered, which increased as the tissue dysplasia worsened [28].

## 3. Studies in Cell Lines

The function of ERβ at the molecular level was investigated in vitro by Hartman et al. They used SW480 colon cancer cells transduced by lentivirus plasmid carrying ERβ; its exogenous over-expression induced a blockage in cell proliferation and cell cycle arrest in the G1 phase, with a reduction in the expression of the oncogene c-Myc (overexpressed in CRC) compared to controls. Following implantation in severe combined immunodeficient/beige mice, a 70% tumor volume reduction was observed in vivo in mice with ERβ re-expression [29]. Similarly, ERβ’s role as a cell cycle modulator was also demonstrated in a previous study using ERα-negative HTC8 colon cancer cells, with the inhibition of proliferation proportional to the level of ERβ expression [30]. Furthermore, there is evidence that ERβ regulates mismatch repair function by microRNA adjustment [31], while estradiol modulates intestinal permeability through ERβ signaling [32].

The potential therapeutic effect of ERβ was investigated by treating HCT116 colon cancer cells with ERβ gene constructed into adenoviral vectors alone or in combination with raloxifene, a selective estrogen receptor modulator. Only the association with raloxifene induced a relevant fall in the proliferation index [33]. In another in vitro study, ERβ acted as an anti-proliferation agent in HCT116 colon cancer cells by impairing the cell cycle but not apoptosis. In detail, ERβ mediated CyclinD1 degradation, thus inhibiting colon cancer cell growth through autophagy. Autophagy took place by suppression of the mammalian target of rapamycin (mTOR) [34]. Finally, treatment with an ERβ-agonist (ERB-041) in three different cell lines (HCT-116, Caco-2, and SW-480) suppressed cysteinyl leukotriene receptor 1, active β-catenin, and COX-2 levels. Furthermore, ERB-041-treated cell lines demonstrated significantly decreased migration and survival, and enhanced apoptotic activity as witnessed by increased caspase-3 and apoptotic blebs [35]. Recently, Indukuri et al. [36] showed that in several cell lines (HT29, SW480) ERβ may bind chromatin, regulating several cystatin genes, and in close proximity of the gene promoter, in both cell lines. Cystatin 5 is indeed a proposed tumor suppressor in CRC.

## 4. Studies in Animal Models

Different studies have demonstrated how estrogens exert their protective effects through an ERβ-dependent mechanism. Weige et al. [37] used ovariectomized wild type and ERβ-knockout mice to assess how E2 supplementation modified cell growth in nonmalignant colonocytes. Results showed that wild type mice had fewer aberrant crypt foci and a higher apoptotic activity when compared to knockout mice, thus demonstrating the relevant protective role of receptor triggering. A significant reduction of apoptotic marker caspase-3, differentiation marker cytokeratin20 and cellular adhesion molecules alpha-catenin and plectin was identified in ERβ-knockout mice compared to wild type littermates, thus highlighting the role of ERβ function as a player in the maintenance of colon epithelial junctions and architecture [38]. In the hypothesis of anti-inflammatory and antitumorigenic effects of estrogen, Son et al. investigated the effects of E2 supplementation in azoxymethane (AOM)/DSS mouse models to assess whether it influenced CRC carcinogenesis [39]. Results showed that mice treated with E2 had lower levels of inflammatory markers and significantly fewer synchronous cancers with an increase in anti-oxidant enzymes, compared to the unsupplemented group. Ovariectomy in the same mouse model did not worsen colitis, but it significantly increased CRC incidence in the proximal colon, which was partially counteracted by E2 supplementation [40]. However, by investigating estrogen’s role in mouse models for colitis-associated cancer, conflicting results were obtained, as the hormone replacement in ovariectomized mice promoted adenomas and invasive cancer formation in both ERα and ERβ knockout mice, compared to placebo [41].

To provide further evidence about the antitumorigenic role of estrogen, treatment with ERβ-selective agonist diarylpropionitrile was administered in male and female Apc^Min/+^ mice; a significant reduction in small intestinal polyp number (39%) and diameter (36%) was seen [42]. Weyant et al. demonstrated a protective role of endogenous estrogen through upregulation of ERβ and a downregulation of ERα in the Apc^Min/+^ mice model; in their study, ovariectomized Min/+ mice with E2 supplementation had an equal number of tumors as littermates that were neither castrated nor supplemented with hormone replacement [43]. Moreover, another study using the same mouse model further suggested that ERβ is an inhibitory modifier of the APC-dependent carcinogenesis in the proximal colon [44]. Estrogen may also regulate the cancer microenvironment, as Jiang suggested. They found that extracellular vesicles treated with estradiol were less capable to induce Tregs in ovariectomized mice, thus counteracting the immunosuppression against cancer. [45].

In the setting of inflammatory bowel diseases, estrogen has also been investigated as a possible modifier of gut microbiota. Colitis and CRC were induced in mice with and without intestine-specific deletion of ERβ (ERβ KO^Vil^) by a single peritoneal injection of AOM followed by administration of DSS, and microbiota was analyzed by sequencing DNA extracted from fecal samples by high-throughput 16S rRNA gene sequencing. Gut microbiota showed reduced diversity in colitis-associated CRC, which decreased further upon lack of ERβ, compared to untreated controls [46].

## 5. Perspective in Humans and Chemoprevention

The previously reported evidence points toward a possible role of estrogen in CRC prevention. Dietary compounds such as the plant-derived phytoestrogens act as estrogenic-like molecules binding to ERs and inducing a similar protective role. Some bind preferentially to ERβ, acting as selective agonists [47]. A preclinical study using phytoestrogen supplementation on Apc^Min/+^ model, which simulates the adenoma-carcinoma sequence pathway of inherited CRC, was carried out by Barone et al. The addition of silymarin, lignin or a combination of both revealed a decrease in the total number of polyps and increased apoptosis compared to control mice (who received a high-fat, low-fiber diet), with the combination of silymarin/lignin achieving the most statistically significant reduction [48]. In a similar study, a combination of silymarin, boswellic acid and curcumin was given to Apc^Min/+^ mice. Those receiving an enriched diet showed fewer polyps, low grade dysplastic areas and carcinomas; furthermore, the supplementation also led to a reduction of cleaved caspase-3 and Cyclin-D1 levels [49]. Following these results, a randomized double-blind placebo-controlled study with an active diet intervention (ADI) with a silymarin-based combination of phytoestrogens and insoluble fibers was performed by the same group. Eligible patients were randomized for ADI or placebo for 60 days before a surveillance colonoscopy for previous sporadic colonic adenomas. It was revealed that 63% of the intervention group had ERERβ protein levels above the median and increased apoptotic markers, evaluated by TUNEL and caspase-3 [50]. A recent study demonstrated in vitro the inhibitory action of silibinin, an active constituent of the phytoestrogen Silymarin, on the β-catenin signaling pathway, a turning point of CRC tumorigenesis [51]. Angiogenesis, another hallmark of cancer, was strongly suppressed by silibinin, associated with the down-regulation of nitric oxide synthase (NOS), cyclooxygenases (COX), hypoxia-inducing factor-1 alpha (HIF-1 alpha) and vascular endothelial growth factor (VEGF) expression [52]. Furthermore, Sameri et al. recently demonstrated a silibinin-induced rise in autophagy and apoptosis [53].

Given their ability to reduce the number of gastrointestinal polyps, phytoestrogens have been studied in high-risk individuals as patients with a FAP diagnosis. After a 3-month ADI with a patented mixture of phytoestrogens and insoluble fibers, a reduction in the number and size of duodenal polyps was achieved in FAP patients with an ileal pouch-anal anastomosis. Histopathological analysis of polyps also highlighted an up-regulation of antitumoral genes as ERβ and MUC2 [54]. In another study using Pirc rats mutated in the APC gene to reproduce FAP pathogenesis, phytoestrogen and insoluble fibers supplementation was proposed as a chemopreventive treatment to postpone prophylactic colectomy due to its capability to counteract colon tumorigenesis [55]. A singular case of a patient with Lynch Syndrome having multiple jejunal polyps has been reported [56]: a three-month supplementation with phytoestrogens led to a reduction of polyp number and size at videocapsule endoscopy.

To date, there is no evidence available showing an association between estrogen receptors and colitis-associated cancer, but some data in murine models suggest a potential role. Indeed, a formulation with silymarin, boswellic acid and curcumin given in AOM/DSS mice led to CRC onset only in the 23.5% versus the 69.6% of controls. The same supplementation promoted ERβ expression and apoptosis in low grade dysplasia areas and counteracted ERα levels [57].

## 6. Conclusions

The role of ERs in CRC is an attractive research question. The current knowledge is greatly based on experimental in vitro and in vivo studies, which in most cases show agreeing results about the protective effect of ERβ on colonic carcinogenesis. Indeed, ERβ stimulation under-regulates colorectal adenomatous polyp growth and modulates relevant pathways in CRC, such as epithelial proliferation/apoptosis balance, mismatch repair function by microRNA adjustment and intestinal permeability control. Based on such assumptions, dietary modulation of ER may be considered as a promising approach, especially in subjects with a genetic predisposition for CRC. Indeed, there are convincing pieces of evidence that patients with inherited polyposis and inflammatory bowel disease could benefit from dietary supplementation with ERβ stimulating agents, such as silymarin. Nevertheless, from a translational medicine point of view, the main limitation of these data is represented by the lack of clinical trials on a large population sample. Currently, only a few case reports or single-center studies on small series have been described. Therefore, the full potential effectiveness of dietary advising for CRC chemoprevention is a field that deserves to be explored by well-designed large-scale studies.
